# Supplementary material for: Empirical methods for controlling false positives and estimating confidence in ChIP-Seq peaks
Source: BMC Bioinformatics. 2008 Dec 5;9:523. doi: 10.1186/1471-2105-9-523 (PMC2628906; doi:10.1186/1471-2105-9-523)
Supplement: Additional File 1 — A variety of html documents from the USeq web site detailing the available applications, their best usage, output file type descriptions, command line menus, etc. [file 1471-2105-9-523-S1.zip › USeqUserGuides/JavaDoc.zip!/JavaDoc/util/bio/annotation/ExonIntron.html]

ExonIntron


|  |  |  |
| --- | --- | --- |
| **Overview** \| **Package** \| **Class** \| **Use** \| **Tree** \| **Deprecated** \| **Index** \| **Help** | |  |
| **PREV CLASS**   **NEXT CLASS** | **FRAMES**    **NO FRAMES**     **All Classes** |  |
| SUMMARY: NESTED \| FIELD \| CONSTR \| METHOD | DETAIL: FIELD \| CONSTR \| METHOD |  |


---


## util.bio.annotation Class ExonIntron

```
java.lang.Object
  util.bio.annotation.ExonIntron
```

**All Implemented Interfaces:**: java.io.Serializable, java.lang.Comparable

---

``` public class ExonIntron extends java.lang.Object implements java.lang.Comparable, java.io.Serializable ```

ExonIntron info.

**See Also:**: Serialized Form

---

**Constructor Summary**

`ExonIntron(Gff3Feature f)`

`ExonIntron(int start,
int end)`

`ExonIntron(int start,
int end,
java.lang.String name)`


| **Method Summary** | |
| --- | --- |
| `int` | `compareTo(java.lang.Object other)` |
| `int` | `getEnd()` |
| `java.lang.String` | `getName()` |
| `java.lang.String` | `getParent()` |
| `int` | `getStart()` |
| `void` | `setEnd(int end)` |
| `void` | `setStart(int start)` |
| `java.lang.String` | `toString()` |

**Methods inherited from class java.lang.Object**

`equals, getClass, hashCode, notify, notifyAll, wait, wait, wait`

**Constructor Detail**

### ExonIntron

```
public ExonIntron(int start,
                  int end)
```

---


### ExonIntron

```
public ExonIntron(int start,
                  int end,
                  java.lang.String name)
```

---


### ExonIntron

```
public ExonIntron(Gff3Feature f)
```


**Method Detail**

### toString

```
public java.lang.String toString()
```

:   **Overrides:**: `toString` in class `java.lang.Object`

---


### compareTo

```
public int compareTo(java.lang.Object other)
```

:   **Specified by:**: `compareTo` in interface `java.lang.Comparable`

---


### getName

```
public java.lang.String getName()
```

---


### getEnd

```
public int getEnd()
```

---


### getStart

```
public int getStart()
```

---


### getParent

```
public java.lang.String getParent()
```

---


### setEnd

```
public void setEnd(int end)
```

---


### setStart

```
public void setStart(int start)
```


---


|  |  |  |
| --- | --- | --- |
| **Overview** \| **Package** \| **Class** \| **Use** \| **Tree** \| **Deprecated** \| **Index** \| **Help** | |  |
| **PREV CLASS**   **NEXT CLASS** | **FRAMES**    **NO FRAMES**     **All Classes** |  |
| SUMMARY: NESTED \| FIELD \| CONSTR \| METHOD | DETAIL: FIELD \| CONSTR \| METHOD |  |


---
